# *Mycobacterial* PE/PPE proteins function as “personal protective equipment” against host defenses

**DOI:** 10.3389/ftubr.2024.1458105

**Published:** 2024-09-18

**Authors:** Carlos Resstel, Bala T. S. A. Madduri, Samantha L. Bell

**Affiliations:** ^1^Center for Emerging and Re-emerging Pathogens, New Jersey Medical School, Rutgers University, Newark, NJ, United States; ^2^Department of Microbiology, Biochemistry & Molecular Genetics, New Jersey Medical School, Rutgers University, Newark, NJ, United States

**Keywords:** secreted effectors, bacterial pathogenesis, virulence factors, macrophages, tuberculosis

## Abstract

*Mycobacterium tuberculosis* (*Mtb*) is the deadliest bacterial infection worldwide, but many molecular details of how it interacts with the innate immune system remain obscure. In particular, although *Mtb* secretes a large number of putative effector proteins, a relatively small number have assigned functions in facilitating host-pathogen interactions. One particularly large family of secreted mycobacterial proteins that remains poorly understood is the PE/PPE proteins. Despite numerous lines of evidence for potential roles in virulence and in mediating host-pathogen interactions, only a small fraction of these 170+ proteins have been well characterized. However, this large family of proteins is likely key for understanding how *Mtb* subverts immune responses, manipulates host cell biology, and establishes a successful infection. Here, we highlight examples of PE/PPEs that have well-defined effects on cell intrinsic pathways in macrophages during mycobacterial infection. Examples include PPE2, which blunts production of reactive oxygen species and nitric oxide; PE_PGRS33, which facilitates bacterial uptake; PE_PGRS29, which directly binds ubiquitin to promote host autophagy and limit pathologic inflammation; MirA, which facilitates actin tail formation to promote cell-to-cell spread; and others. Understanding the full spectrum of PE/PPE functions is critical for understanding *Mtb* pathogenesis and for developing new strategies to combat the worldwide TB pandemic. Advancing the lagging research efforts characterizing this mysterious family of effector proteins is critical for the TB field.

## Introduction

Despite worldwide efforts to end the tuberculosis (TB) pandemic, around 10 million people fall ill with TB annually, making TB the leading cause of death from an infectious disease (1). While TB is a treatable infection, the alarming rise of drug resistant strains (1–4), the lack of a highly protective vaccine (5, 6), and the shortage of reliable point-of-care diagnostics (7, 8) make the development of new therapeutics an urgent necessity. To develop new, effective TB treatments, we need a precise understanding of the molecular mechanisms of TB pathogenesis.

TB is caused by the bacterium *Mycobacterium tuberculosis* (*Mtb*), an expert pathogen capable of evading and manipulating the immune response for its benefit. Once infectious droplets are inhaled by a new host, the bacterium reaches the lungs and is phagocytosed by surveilling alveolar macrophages (9, 10). After engulfment by these macrophages, *Mtb* uses its ESX-1 secretion system to permeabilize its phagosome, gain access to the macrophage cytosol, and modulate the host cell (11, 12). Using a wide array of lipids and secreted proteins called effector proteins, *Mtb* manipulates host cell biology to block antibacterial pathways and create an intracellular replicative niche (13–15).

One class of *Mtb* effector proteins are the PE/PPE proteins, named for the proline-glutamic acid (PE) or proline-proline-glutamic acid (PPE) motifs in their N-terminal domains. Their C-terminal domains vary widely but commonly contain disordered domains or domains predicted to interface with host biology. PE/PPEs were first identified upon the complete sequencing of the *Mtb* genome (16, 17). Strikingly, this study showed that ~10% of the *Mtb* genome coding capacity is dedicated to PE/PPEs (16). The sheer genomic volume dedicated to these proteins, compounded by their expansion specifically in pathogenic mycobacterium, suggests they play a critical role in virulence. For example, the non-pathogenic species *M. smegmatis* has approximately 10 PE/PPE genes while the pathogens *M. bovis* and *M. marinum* have approximately 160 and 280 PE/PPEs, respectively (18–20). Several PE/PPE proteins are also associated with the genomic loci encoding *Mtb*'s five ESX (type VII) secretion systems, which are tightly associated with virulence (19, 21, 22). Furthermore, even the dramatically reduced genome of the leprosy-causing bacterium *M. leprae* has 12 potential PE/PPE genes (20, 23), again pointing to the important role PE/PPEs play in mycobacterial pathogenesis.

**Figure 1:**
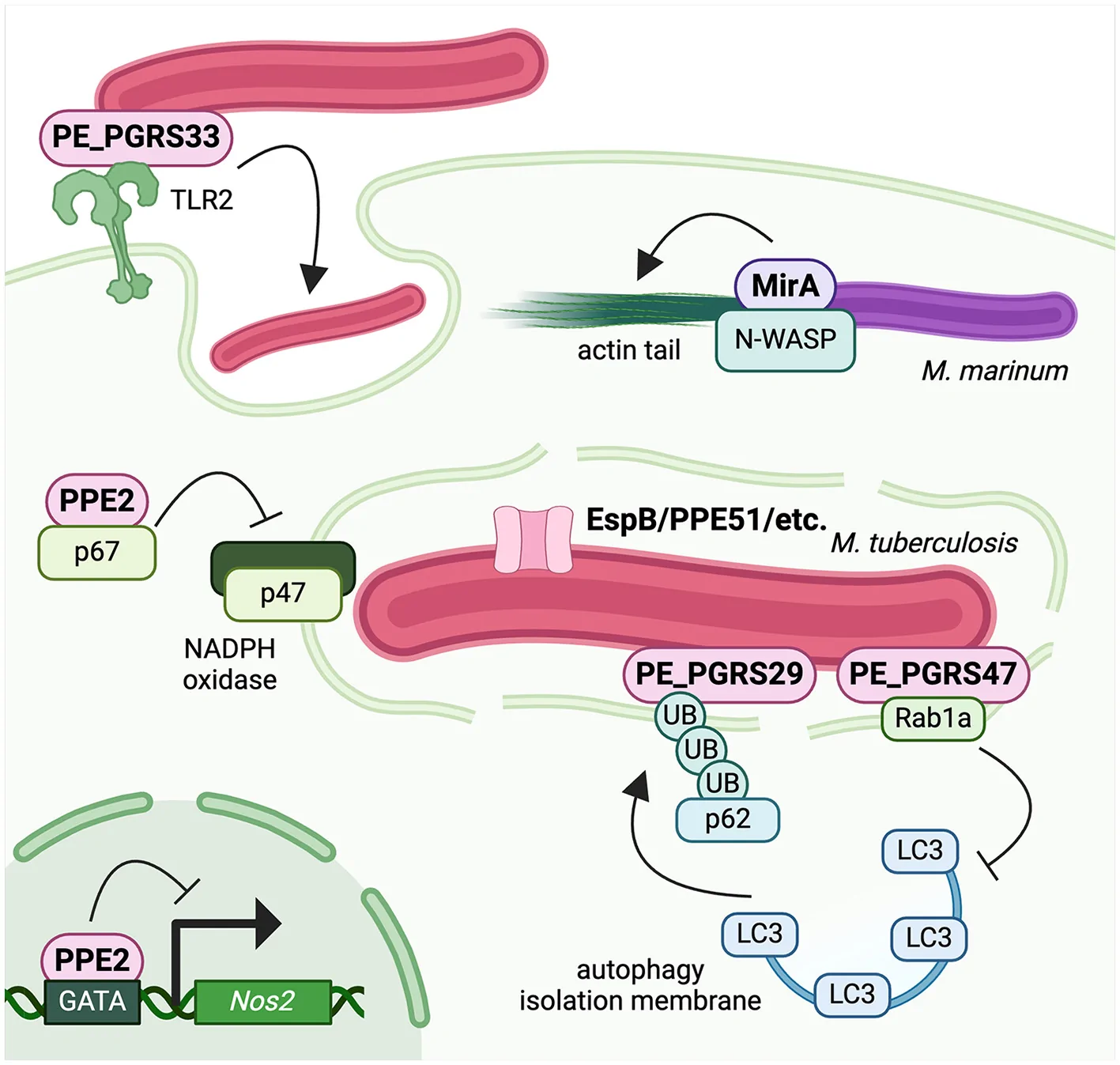
Mycobacteria use PE/PPE proteins to hijack host cell biology and evade antibacterial defenses. PE_PGRS33 is surface exposed and promotes *Mtb* uptake by macrophages. PPE2 binds p67^phox^ to inhibit assembly of phagosomal NADPH oxidase and blunt ROS production, and it also translocates into the nucleus to block *Nos2* transcription and blunt NO production. PE_PGRS29 binds polyubiquitin chains to enhance targeting of *Mtb* to autophagy and regulate inflammatory responses, while PE_PGRS47 binds to Rab1a to block autophagy induction and formation of the isolation membrane. The *M. marinum* PGRS protein MirA recruits the actin nucleator N-WASP to the bacterial surface to polymerize actin into tails for cell-to-cell spread. Created with BioRender.com.

In this mini review, we discuss a subset of PE/PPE proteins that act as virulence factors during *Mtb* infection (Figure 1). By highlighting examples of especially well-characterized PE/PPEs that have a strong combination of microbiology and cell biology evidence to support their roles during infection, we aim to demonstrate the evidence necessary to establish PE/PPEs as bona fide mycobacterial virulence factors. Moreover, we hope to emphasize the importance of PE/PPEs in mediating *Mtb* virulence and underscore the need to define the molecular functions of the remaining PE/PPEs, many of which still have poorly defined roles in *Mtb* infection.

## PE/PPE proteins

Broadly speaking, the N- and C-terminal domains of PE/PPE proteins are predicted to carry out divergent functions, with the N-terminus facilitating secretion and the C-terminus exerting biological functions (24, 25). The N-termini of PE and PPE proteins contain alpha helices that form hydrophobic faces through which they interact as heterodimers (26–28). These heterodimers are bound by the chaperone EspG, which delivers them to an ESX secretion system for export (26–30). The C-termini of PE/PPE proteins, on the other hand, are predicted to carry out biological functions, whether in bacterial physiology or in mediating host interactions. The PGRS regions that define the PE_PGRSs are the best characterized PE/PPE domains due to their documented ability of many to alter host cell biology. For example, PE_PGRS5's C-terminal PGRS domain drives the protein's ER localization and ability to induce ER stress regardless of the specific PE domain that is fused to it (31). Similarly, the PGRS domain in the *M. marinum* PE_PGRS protein MMAR_0242 prevents phagosome-lysosome fusion in macrophages and is both required for intracellular *M. marinum* replication and sufficient to enhance *M. smegmatis* survival (32). The theme of PE/PPEs' C-terminal domains driving their virulence functions holds true for a vast majority of characterized PE/PPEs and is discussed in detail below for the functional C-terminal domains of PPE2, PE_PGRS33, and MirA.

### PPE2

Because PPE2 has been identified in culture supernatants, it is categorized as a secreted protein (33), and studies have sought to identify its function in host cells. One study ectopically expressed PPE2 in macrophages to assess how it affected host cell biology and found that PPE2 expression decreased nitric oxide (NO) production upon LPS stimulation (34). Importantly, another study demonstrated that macrophages infected with ΔPPE2 *Mtb* produce higher levels of NO (33), further indicating that PPE2 blunts this critical antibacterial response. PPE2 has also been tied to NO production via its nuclear localization and impact on gene expression. PPE2 possesses a nuclear localization signal (NLS) that binds to importin proteins, which mediate nuclear trafficking (34). The authors demonstrated that through its NLS, PPE2 localizes to the nucleus where it binds to the GATA region of the *Nos2* promoter (34). As a result, PPE2 represses *Nos2* expression and NO production by macrophages, restraining their antibacterial capacity (34).

PPE2 is also required to dampen the oxidative burst during *Mtb* infection (35). Macrophages infected with ΔPPE2 *Mtb* generated more reactive oxygen species (ROS) compared to those infected with wild-type *Mtb* (35). This was further supported by the finding that macrophages ectopically expressing PPE2 had lower ROS generation (35). In determining the mechanism for PPE2-dependent ROS differences, the authors of this study found that PPE2-expressing macrophages had reduced recruitment of p47^phox^ and p67^phox^ to the phagosome and decreased NADPH oxidase activity in phagosome-containing membrane fraction (35). Furthermore, the authors identified an interaction between PPE2 and p67^phox^, which is thought to prevent p67^phox^ recruitment to the phagosome and assembly of the NADPH oxidase complex. The authors also determined that a specific residue in the C-terminal domain of PPE2, W263, was required for PPE2′s interaction with p67^phox^, and therefore, its ability to blunt ROS production (35). Together, these studies suggest that individual PE/PPE proteins can affect more than one pathway via more than one mechanism to effectively subvert macrophage defenses.

### PE_PGRS33

PE_PGRS33 localizes to the mycobacterial cell wall and elicits an immune response, indicating it interacts with host cellular machinery during *Mtb* infection (36–38). In a study examining the entry of a ΔPE_PGRS33 *Mtb* mutant, PE_PGRS33 was found to be required for uptake specifically in macrophages but dispensable for *Mtb*'s intracellular replication of *Mtb* (39). Because pathogens are known to hijack TLR2 signaling for uptake into immune cells, the authors examined the requirement of TLR2 for *Mtb*'s PE_PGRS33-dependent uptake. They found that blocking TLR2 through genetic or chemical means dramatically decreased macrophages' uptake of wild-type *Mtb* (39). By complementing the knockout with several PE_PGRS33 truncations, the authors found that the C-terminal PGRS domain, and particularly amino acids 140–260, was required for mediating TLR2-dependent uptake (39). Another study exploring the intracellular function of PE_PGRS33 found that when ectopically expressed in T cells, PE_PGRS33 colocalizes with mitochondria and induces apoptosis (40). This observation is supported by a study using purified PE_PGRS33 and PE_PGRS33 ectopically expressed in *M. smegmatis* that similarly found PE_PGRS33 induces cell death in macrophages (41). Interestingly, despite these cell biology phenotypes, ΔPE_PGRS33 *Mtb* exhibits no defects in intracellular replication or growth defects in a mouse infection model (39, 42). However, deletion of PE_PGRS33 leads to enhanced pathogenesis during the chronic stage of infection (42), suggesting that uptake via TLR2 is key for *Mtb* pathogenesis.

Clinical isolates of *Mtb* commonly harbor polymorphisms in the PE_PGRS33 gene, and many of these mutations result in frameshifts and truncations in the PGRS domain (43, 44). In a study exploring the ramifications of these clinical mutations, the authors found that complementing ΔPE_PGRS33 *Mtb* with clinical variants of PE_PGRS33 did not rescue the mutant's defect in uptake by macrophages (42). Moreover, complemented strains exhibited more extracellular bacilli in murine lungs and induced more tissue damage at later time points post-infection (42). Together, these studies suggest that PE_PGRS33 promotes *Mtb*'s uptake by macrophages and loss of this gene, as is frequently observed in clinical strains, may promote *Mtb* pathogenesis.

### PE_PGRS47

Interest in PE_PGRS47 initially stemmed from its identification in a screen identifying *Mtb* factors that inhibit antigen presentation, which is a major virulence strategy employed by *Mtb* (45). The authors of this study introduced an *Mtb* cosmid library into *M. smegmatis* and identified bacterial clones that decreased MHC-II antigen presentation in dendritic cells. The authors found that expression of PE_PGRS47 in *M. smegmatis* not only decreased antigen presentation but also enhanced intracellular bacterial survival (45). To explain these two phenotypes, the authors measured autophagy, which is a potent antibacterial response initiated by macrophages to control intracellular *Mtb* infection. In macrophages infected with ΔPE_PGRS47 *Mtb*, there was increased autophagy induction and more *Mtb* surrounded by autophagy machinery (45). Critically, the authors also demonstrated that ΔPE_PGRS47 *Mtb* was attenuated in a mouse model of infection (45).

Because the mechanism of how PE_PGRS47 inhibits autophagy was unknown, a subsequent study investigated this by ectopically expressing PE_PGRS47 in macrophages. The authors found that expression of PE_PGRS47 blocked the induction of autophagy and promoted bacterial survival (46). Using immunoprecipitation paired with mass spectrometry, the authors determined this was due to PE_PGRS47′s interaction with Rab1a, which the authors found to be required for autophagy induction during *Mtb* infection (46). The mechanism by which PE_PGRS47 may block recruitment of autophagy machinery to *Mtb* remains to be determined, but decreased LC3 recruitment to *Mtb* may arise from Rab1A's role in elongating the isolation membrane (46). In a related study, the same authors identified five PE/PPEs in addition to PE_PGRS47 (PE_PGRS21, PE_PGRS30, PPE44, and PPE51) that are required for *Mtb*'s intracellular replication and for inhibiting bulk autophagy (47). However, the specific mechanisms by which each inhibits autophagy during *Mtb* infection, including the domains required for these functions, remain to be defined.

### PE_PGRS29

Interaction between a PE/PPE and the same autophagy pathway but with opposite consequences is mediated via PE_PGRS29 (48). PE_PGRS29 is a surface-exposed PE protein that contains a ubiquitin-associated (UBA) domain, which directly interacts with polyubiquitin chains (48). The interaction between PE_PGRS29 and ubiquitin recruits downstream selective autophagy machinery, including p62 and LC3, which targets *Mtb* to xenophagy for bacterial clearance (48). The authors studied ΔPE_PGRS29 *Mtb* and found that in macrophages, the deletion mutant recruits less ubiquitin, p62, and LC3, and is better able to survive and replicate intracellularly (48). While recruiting autophagy machinery enhances *Mtb* killing, the authors argue this is a cost that may be outweighed by the substantial benefit of dampening host cell responses and preventing excessive inflammation in order to prolong infection and establish latency. Indeed, mice infected with ΔPE_PGRS29 *Mtb* exhibited worse immunopathology, including larger lung lesions, more cellular infiltrates in the lung, and increased expression of proinflammatory cytokines (48). Intriguingly, PE_PGRS29 represents a rare example where the functional domain/residue resides in the PE domain (L65) rather than in the PGRS domain (48). Whether the PGRS domain of PE_PGRS29 plays additional roles interfacing with host cell biology remains to be determined.

### MirA

*M. marinum*, which is primarily a fish pathogen but also causes infection in humans, exhibits actin-based motility within infected cells to enable its cell-to-cell spread. However, the molecular mechanism governing this virulence strategy was unknown. In a recent study using transposon mutagenesis and fluorescence microscopy, the authors identified the PE_PGRS gene responsible for *M. marinum*'s actin tail formation (MMAR_3581) and named it mirA (mycobacterial intracellular rocketing A) (49). The authors found that MirA interacts with the host actin nucleator N-WASP to induce actin polymerization, and while MirA is not required for intracellular growth, it is required for *M. marinum* to form actin tails and spread from cell to cell (49). Interestingly, ectopic expression of MirA in host cells induces the formation of actin tails on lipid droplets, mirroring the actin tails on intracellular bacilli (49). An amphipathic helix within MirA's PGRS domain is required for MirA to promote actin-based motility and for MirA to localize to lipid droplets, but this helix is dispensable for MirA's interaction with N-WASP (49). These findings indicate that this helix anchors MirA in the mycobacterial cell wall where it recruits N-WASP to polymerize actin and propel the bacterium through the cytoplasm. Importantly, the authors identified similar amphipathic helices in many PGRS domains (49), suggesting it may be a common feature of PE_PGRS proteins wherein they are tethered to the bacterial surface to interface with host cell machinery.

## Additional functions of PE/PPE proteins

Upon the initial discovery of PE/PPEs, they were hypothesized to play a role in immune evasion via antigenic variation due to their highly repetitive gene structures (50). This is a common strategy used by pathogens in which they alter their cell surfaces to make them unrecognizable to the host's adaptive immune system. This hypothesized role for PE/PPEs is supported by studies identifying PE/PPE antibodies in infected hosts (38, 51, 52), which indicates the adaptive immune system does recognize this protein family and suggests an evasion mechanism would be beneficial to the bacterium. However, studies have shown that antibodies capable of detecting PE/PPEs usually target their conserved N-terminal region rather than their highly variable and repetitive C-termini (38, 53). Therefore, while a role in antigenic variation has not been ruled out, it appears the host develops broadly neutralizing antibodies rather than antibodies against antigens undergoing antigenic shift or drift.

A growing body of evidence also indicates that a subset of PE/PPEs act as channels to transport nutrients and even protein dimers across the especially thick mycomembrane (54). In most cases, PE proteins appear to help export PPE proteins to the outer membrane where PPEs function independently. For example, PPE51 export to the outer membrane requires PE19 (55), and once there, PPE51 promotes phosphate and disaccharide uptake (55, 56). Likewise, PE20 promotes export of PPE31 to facilitate magnesium uptake (55). Similarly, PPE36 and PPE62 are anchored to the outer cell membrane and directly bind to heme, playing a role in iron acquisition, and PPE36 transport to the outer membrane has been shown to be facilitated by PE22 (57). Conclusively determining whether porin-like structures are formed exclusively by PPEs or by complexes of PE/PPEs has been challenging since most studies examining porin function have relied on genetic approaches. Moreover, how PPE porins contribute to *Mtb* physiology during infection and whether they can be considered bona fide virulence factors is not well established.

Recent reports have also provided compelling evidence demonstrating that PE/PPE proteins themselves form channels in the mycomembrane to facilitate their own secretion. Two recent studies used cryo-electron microscopy to determine the structure of EspB, a noncanonical PE/PPE protein that contains both PE and PPE domains and is cleaved in the periplasm (58, 59). These studies found that EspB forms a large channel capable of accommodating folded proteins, including PE/PPE heterodimers (58, 59). Moreover, the authors found that channel-like structures were only formed by EspB proteins from slow-growing mycobacteria (58), suggesting EspB channels may play a critical role in pathogenesis. These studies help clarify the mechanism by which PE/PPEs form channels or porins and illustrate how PE/PPEs may be secreted into the extracellular space where they interact with the host cell.

## Perspectives

Here we have highlighted PE/PPE proteins with well-defined host targets and well-characterized virulence roles during mycobacterial infection (Figure 1). For a vast majority of these studies, authors have identified an interaction with a host protein, determined the region of the PE/PPE required for its function, discovered a cell biology phenotype, and demonstrated that an *Mtb* mutant exhibits a similar phenotype during infection. While addition of purified PE/PPEs to host cells and ectopic expression of PE/PPEs in *M. smegmatis* provides useful initial data on possible virulence functions, ectopic expression in macrophages and infections with *Mtb* knockout and complemented strains has provided robust physiological data on PE/PPE functions during infection.

The most well-characterized PE/PPEs belong to the human pathogen *Mtb* due to its large public health impact. However, other mycobacteria express numerous diverse PE/PPE proteins with the potential to provide novel insight into mycobacterial virulence strategies. *M. marinum*'s MirA is a prime example as it revealed that many PGRS proteins possess amphipathic helices that likely facilitate tethering to the mycobacterial surface (49). Exploration of PE/PPEs from non-tuberculous mycobacteria (NTM), such as *M. abscessus*, will be critical for the development of new therapeutic interventions for these emerging pathogens that are even more challenging to successfully treat than *Mtb*. Additionally, since PE/PPEs are primarily studied in the context of pathogenesis, the small subset of non-virulence related PE/PPEs has been underexplored and may hold valuable insights into mycobacterial physiology.

One major motivation for studying PE/PPEs as potential virulence factors lies in their significant variability between *Mtb* strains and lineages (60, 61). For example, the hypervirulent Beijing strains in lineage 2 have a mutation in the gene encoding PPE38 that prevents its secretion (62). Because PPE38 assists in the secretion of ESX-5-dependent PE/PPEs, this mutation in PPE38 renders Beijing strains defective for secretion of many PE/PPEs (62). Interestingly, this secretion defect increases the virulence of Beijing strains, and introducing wildtype PPE38 reverts these strains' hypervirulence phenotypes (62), suggesting that PE/PPEs can not only drive strain-specific virulence phenotypes but also play complex roles mediating *Mtb* pathogenesis. Together with the studies reviewed above, this illustrates how *Mtb* has evolved to carefully balance and fine tune the use of effectors to both subvert and exploit host defenses for its benefit.

In reviewing a handful of well-characterized PE/PPEs, we have highlighted the challenge ahead performing detailed characterization of the many remaining weakly characterized PE/PPE proteins. Future efforts should focus on identifying precise mechanisms by which individual PE/PPEs promote mycobacterial infection, including performing structural, biochemical, molecular, and cellular studies. Elucidating the roles of these 200+ proteins will deepen our understanding of mycobacterial pathogenesis and help identify novel targets and intervention strategies for future TB treatments.
